# Twofold correlation spreading in a strongly correlated lattice Bose gas

**DOI:** 10.1038/s41598-019-40679-3

**Published:** 2019-03-11

**Authors:** Julien Despres, Louis Villa, Laurent Sanchez-Palencia

**Affiliations:** CPHT, Ecole Polytechnique, CNRS, Institut Polytechnique de Paris, F-91128 Palaiseau, France

## Abstract

We study the spreading of correlations in the Bose-Hubbard chain, using the time-dependent matrix-product state approach. In both the superfluid and the Mott-insulator phases, we find that the time-dependent correlation functions generally display a universal twofold cone structure characterized by two distinct velocities. The latter are related to different microscopic properties of the system and provide useful information on the excitation spectrum. The twofold spreading of correlations has profound implications on experimental observations that are discussed.

## Introduction

In the last decades, simultaneous progress of the many-body quantum theory and the experimental control of quantum matter in condensed matter and atomic, molecular, and optical physics has given dramatic momentum to the understanding of the out-of-equilibrium dynamics of correlated quantum systems^1–10^. The spreading of quantum correlations governs many fundamental phenomena, including the propagation of information and entanglement, thermalization, and the area laws for entanglement entropy. For lattice systems with local interactions, the existence of Lieb-Robinson (LR) bounds implies the emergence of a causal light cone beyond which the correlations are exponentially suppressed^11–13^. So far, light-cone-like spreading of correlations has been reported in short-range interacting models^14–17^ as well as long-range models^18–27^ where weaker LR bounds exist^13,28^. However, many questions remain open. For instance, it is still debated whether a non-linear cone emerges in generic long-range systems, for which different results point towards either super-ballistic, ballistic or sub-ballistic spreading. It was recently proposed that these apparently conflicting results can be reconciled by the coexistence of several signals governed by different scaling laws^27^. This behavior may be related to the non-linearity of the quasiparticle excitation spectrum, and may also appear in systems with short-range interactions. In the later case, it is expected that both signals spread ballistically but with different velocities. However, this picture relies on mean-field theory, which ignores potentially important dynamical effects, such as quasiparticle collisions and finite lifetime.

In this work, using an exact many-body approach beyond mean-field theory, we demonstrate the emergence of a universal twofold dynamics for the spreading of correlations in a generic short-range, strongly correlated quantum model. Specifically, we consider the one-dimensional Bose-Hubbard model and use time-dependent tensor network techniques based on matrix product states. Spanning the phase diagram, we generally find a twofold structure of the space-time correlation pattern, characterized by two distinct velocities, essentially irrespective of the correlation function. Exceptions of this twofold structure are discussed below. In the superfluid mean-field regime and in the Mott insulator phase, the two velocities associated to the correlation spreading are readily interpreted from the properties of the corresponding excitation spectrum. In the strongly correlated superfluid regime, the sound velocity is known but not the full excitation spectrum. There, our results show beyond Luttinger liquid behavior and provide useful information about the excitation spectrum beyond the phonon branch. The emergence of a universal twofold spreading of correlations has profound implications on experimental observations, which we discuss, including with a view towards extensions to long-range systems.

## Model and Approach

**Figure 1:**
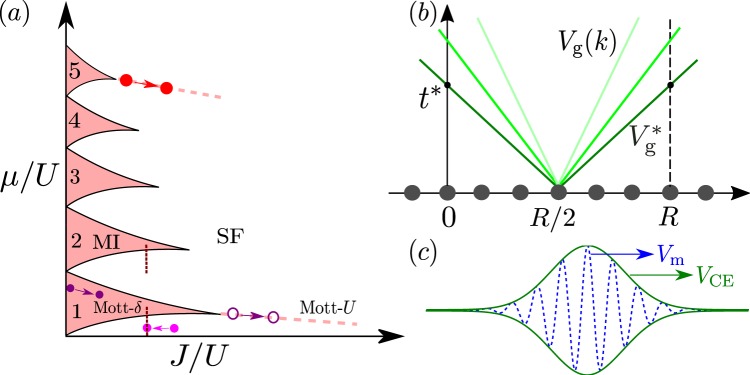
Quantum quench in the Bose-Hubbard model. (**a**) Schematic phase diagram as a function of the inverse interaction strength and chemical potential, comprising a MI phase (pink lobes at integer fillings $\bar{n}$) and a SF phase. The Mott-*U* transition at unit filling is indicated by the dashed pink line and the Mott-*δ* transition by the vertical line. The arrows indicate the various quenches considered in this work. (**b**) Generation of correlations between two points at a distance *R* by pairs of counter-propagating quasiparticles emitted at the mid-point *R*/2. The first correlation is generated by the fastest quasiparticles at the activation time ${t}^{\ast }=R/2{V}_{{\rm{g}}}^{\ast }$. (**c**) Correlation spreading in the vicinity of the correlation edge (CE). The correlation function forms a periodic series of maxima moving at the velocity ${V}_{{\rm{m}}}=2{V}_{\phi }^{\ast }$, with an envelope moving at the velocity ${V}_{{\rm{CE}}}=2{V}_{{\rm{g}}}^{\ast }$.

The Hamiltonian of the one-dimensional (1D) Bose-Hubbard (BH) model, considered throughout this work, reads as

1 $$\hat{H}=-\,J\,\sum _{R}\,({\hat{a}}_{R}^{\dagger }{\hat{a}}_{R+1}+{\rm{h}}.{\rm{c}}.)+\frac{U}{2}\,\sum _{R}\,{\hat{n}}_{R}({\hat{n}}_{R}-1),$$

where ${\hat{a}}_{R}$ and ${\hat{a}}_{R}^{\dagger }$ are the bosonic annihilation and creation operators on site *R*, ${\hat{n}}_{R}={\hat{a}}_{R}^{\dagger }{\hat{a}}_{R}$ is the occupation number (filling), *J* is the hopping amplitude, *U* > 0 is the repulsive on-site interaction energy, and the lattice spacing is fixed to unity ($R\in {\mathbb{Z}}$). At equilibrium and zero-temperature, the phase diagram of the 1D BH model is well known^29,30^, and sketched on Fig. 1(a). It comprises a superfluid (SF) and a Mott insulator (MI) phase, determined by the competition of the hopping, the interactions, and the average filling $\bar{n}$ (or, equivalently, the chemical potential *μ*). For commensurate filling, $\bar{n}\in {{\mathbb{N}}}^{\ast }$ the SF-MI (Mott-*U*) transition is of the Berezinskii-Kosterlitz-Thouless type, at the critical value ${u}_{c}\simeq 3.3$ for unit filling ($\bar{n}=1$) in 1D^31–34^. For incommensurate filling, the Bose gas is a SF for any value of *U*/*J*. The commensurate-incommensurate (Mott-*δ*) transition, of the mean-field type, is then driven by doping when $\bar{n}$ approaches a positive integer value for sufficiently strong interactions.

We study the out-of-equilibrium dynamics of the BH model by applying a sudden global quench^14,16,17,35–40^, as can be realized in ultracold-atom experiments^15,41–43^. We start from the ground state for some initial value of the interaction parameter (*U*/*J*)_0_ and let the system evolve with a different value of *U*/*J*. In the following, we consider a variety of quenches spanning the phase diagram, see arrows on Fig. 1(a). We study the spreading of the phase and density fluctuations, via the connected correlation functions ${G}_{1}(R,t)=\langle {\hat{a}}_{R}^{\dagger }(t){\hat{a}}_{0}(t)\rangle -\langle {\hat{a}}_{R}^{\dagger }(0){\hat{a}}_{0}(0)\rangle$ and ${G}_{2}(R,t)=g(R,t)-g(R,0)$ with $g(R,t)=\langle {\hat{n}}_{R}(t){\hat{n}}_{0}(t)\rangle -\langle {\hat{n}}_{R}(t)\rangle \langle {\hat{n}}_{0}(t)\rangle$. Both observables can be measured in experiments using time-of-flight and fluorescence microscopy imaging, respectively^15,42–44^.

All the results presented below are obtained using density-matrix renormalization group simulations within the time-dependent matrix-product state (*t*-MPS) representation^45–47^. A careful analysis of the numerical cut-offs (high-filling cut-off and MPS bond dimension) has been systematically performed to certify the convergence of the results in all the considered cases. This is particularly critical for quenches in the SF phase where the numerical requirements are most binding (For further details, see Supplemental Material. It contains information about the *t*-MPS calculations, the spreading of the one-body correlations (*G*_1_) in the meanfield regime and two-body correlations (*G*_2_) in the deep MI phase, as well as the mapping onto the Lieb-Liniger model).

## Mean-Field Regime

**Figure 2:**
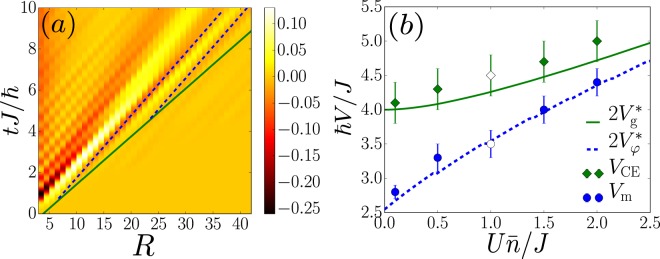
Spreading of correlations in the mean-field regime, see red arrow on Fig. 1(a). (**a**) *t*-MPS result of *G*_2_(*R*, *t*) for a quench to *U*/*J* = 0.1, together with ballistic fits to the CE (solid, green line) and minima (dashed, blue lines). (**b**) Velocities of the CE (*V*_CE_, green diamonds) and minima (*V*_m_, blue disks), found from the fits, versus the interaction strength, and comparison to twice the group velocity $2{V}_{{\rm{g}}}^{\ast }$ (solid green line) and twice the phase velocity $2{V}_{\phi }^{\ast }$ (dashed blue line). All the quenches are performed with $\bar{n}=5$ from ${(U/J)}_{0}=0.2$, except for the points at $U\bar{n}/J=1$ where *U*/*J* = 0.2 and we use a different initial value, (*U*/*J*)_0_ = 0.4 (open points).

We first consider the mean-field regime in the SF phase, where the numerical results can be compared to analytic predictions. This regime is characterized by a small Lieb-Liniger parameter, $\gamma \equiv U/2J\bar{n}\ll 1$. Figure 2(a) displays the *t*-MPS result for the *G*_2_ correlation function versus distance (*R*) and time (*t*) for a quench from ${(U/J)}_{0}=0.2$ to $U/J=0.1$ and $\bar{n}=5$, *i*.*e*. from ${\gamma }_{0}=0.02$ to $\gamma =0.01$ [see red arrow on Fig. 1(a)]. It clearly shows a spike-like structure, characterized by two different velocities. On the one hand, a series of parallel maxima and minima move along straight lines corresponding to a constant propagation velocity *V*_m_ (the dashed blue lines show fits to two of these minima). On the other hand, the various local extrema start at different activation times ${t}^{\ast }(R)$. The latter are aligned along a straight line with a different slope (solid green line), corresponding to a constant velocity *V*_CE_. The latter defines the correlation edge (CE) beyond which the correlations are suppressed. Similar results are obtained for all the other quenches in the mean-field regime, as well as for the *G*_1_ function (Note that the signal for *G*_1_ is, however, less sharp than for *G*_2_. This may be attributed to the long-range correlations present in the initial state, which partially blur the CE (For further details, see Supplemental Material. It contains information about the *t*-MPS calculations, the spreading of the one-body correlations (*G*_1_) in the meanfield regime and two-body correlations (*G*_2_) in the deep MI phase, as well as the mapping onto the Lieb-Liniger model)).

This twofold structure near the CE is readily interpreted using the quasiparticle picture, which we briefly outline here (for details, see ref.^27^): the *G*_1_ and *G*_2_ correlation functions are expanded onto the elementary excitations of the system. In the mean-field regime of the BH model, the latter are Bogoliubov quasiparticles with quasimomenta $k\in [\,-\,\pi ,+\,\pi ]$ and dispersion relation

2 $${E}_{k}\simeq \sqrt{{\varepsilon }_{k}({\varepsilon }_{k}+2\bar{n}U)},$$

where ${\varepsilon }_{k}=4J\,{\sin }^{2}(k/2)$ is that of the free-particle tight-binding model. A correlation between two points at a distance *R* is seeded when two correlated, counter-propagating quasiparticles emanating from the center reach the two points, see Fig. 1(b). The fastest ones are those with the maximum group velocity, ${V}_{{\rm{g}}}^{\ast }=\mathop{{\rm{\max }}}\limits_{k}({\hslash }^{-1}\partial {E}_{k}/\partial k)$. It yields the activation time ${t}^{\ast }(R)=R/2{V}_{{\rm{g}}}^{\ast }$ and the CE velocity ${V}_{{\rm{CE}}}=2{V}_{{\rm{g}}}^{\ast }$, consistently with the expected Lieb-Robinson bound^11,35^. More precisely, the correlation at a distance *R* and a time *t* is built from a coherent superposition of the contributions of the various quasiparticles. In the vicinity of the CE, only the fastest quasiparticles, *i*.*e*. those with a quasimomentum *k* close to ${k}^{\ast }$, contribute. It creates a sine-like signal at the driving spatial frequency ${k}^{\ast }$, whose extrema move at twice the phase velocity ${V}_{\phi }(k)={\hslash }^{-1}{E}_{k}/k$ with $k={k}^{\ast }$, *i*.*e*. ${V}_{{\rm{m}}}=2{V}_{\phi }^{\ast }$^27^. The dispersion around ${k}^{\ast }$ then modulates the sine-like signal by an envelope moving at the CE velocity *V*_CE_, see Fig. 1(c). This behavior is reminiscent of the propagation of a coherent wave packet in a dispersive medium^48–50^. Indeed, a narrow-band wave packet centered around the driving spatial frequency *k*_0_ propagates at the group velocity *V*_g_(*k*_0_) while the maxima move at the phase velocity ${V}_{\phi }({k}_{0})$. In a dispersive medium, the spectrum is non linear hence these two velocities differ in general. In the case we consider here, the value *k*_0_ = k* is selected by the onset in the vicinity of the correlation cone. To test this picture quantitatively, we have extracted the velocities *V*_m_ and *V*_CE_ from the *t*-MPS results for *G*_2_(*R*, *t*) by tracking the local extrema and the activation time respectively. The results, displayed on Fig. 2(b), show excellent agreement with the theory, *i*.*e*. ${V}_{{\rm{CE}}}\simeq 2{V}_{{\rm{g}}}^{\ast }$ and ${V}_{{\rm{m}}}\simeq 2{V}_{\phi }^{\ast }$ within the fitting errorbars. This cross-validates the *t*-MPS results in the most-demanding SF, mean-field regime on the one hand and the quasiparticle picture above on the other hand. Note that the *t*-MPS results are numerically exact and include effects beyond the Bogoliubov approximation, such as quasiparticle collisions.

## Strongly Correlated Regime at Unit Filling

**Figure 3:**
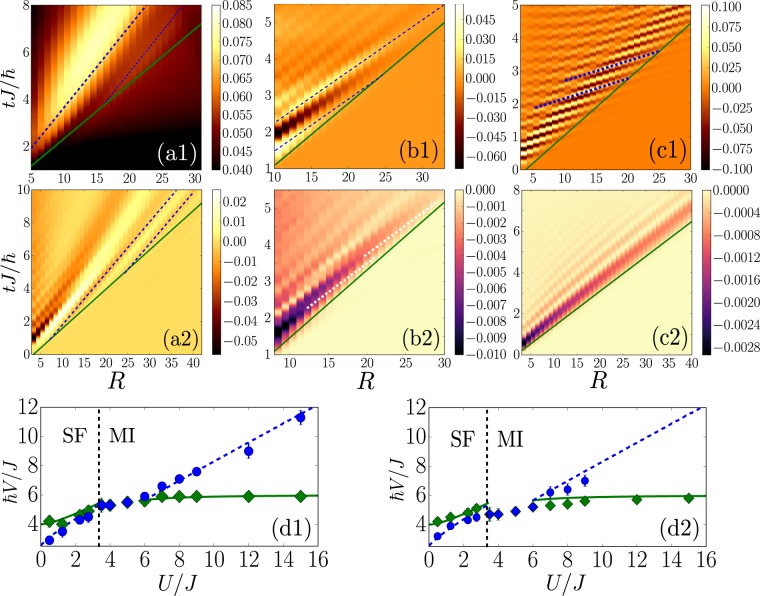
Spreading of the *G*_1_ (upper row) and *G*_2_ (lower row) correlations in both the SF and MI phases for $\bar{n}=1$, scanning the after-quench interaction *U*/*J* along the Mott-*U* transition, see pink dashed line and magenta arrows on Fig. 1(a): (**a**) SF regime with *U*/*J* = 0.5; (**b**) MI regime near the critical point with *U*/*J* = 8; (**c**) deep MI regime with *U*/*J* = 24. The solid green and dashed blue lines correspond to fits to the CE and extrema, respectively. Note that on panel (b2), the fits to the maxima are shown as dashed white lines for clarity. (**d**) Spreading velocities *V*_CE_ (green diamonds) and *V*_m_ (blue disks), as extracted from fits to the *t*-MPS data, and comparison to the characteristic velocities $2{V}_{{\rm{g}}}^{\ast }$ (solid green lines) and $2{V}_{\phi }^{\ast }$ (dashed blue lines), as found from the dispersion relation in the SF [Eq. (2)] and MI [Eq. (3)] regimes. All the quenches are performed from the initial values (*U*/*J*)_0_ = 1 for the SF regime and (*U*/*J*)_0_ = ∞ for the MI regime.

We now turn to the strongly correlated regime *γ* ~ 1, where the correlation functions cannot be systematically computed. We first scan the after-quench interaction parameter *U*/*J* from the SF to the MI, along the Mott-*U* transition at unit filling [$\bar{n}=1$, see magenta arrows on Fig. 1(a)]. Note that each quench is performed in a unique phase: for $U/J < {u}_{{\rm{c}}}\simeq 3.3$ (SF regime), we use the initial interaction strength ${(U/J)}_{0}=1$ while for $U/J > {u}_{{\rm{c}}}$ (MI regime), we start from ${(U/J)}_{0}=\infty$. Figure 3 shows typical results for the spreading of the *G*_1_ (upper row) and *G*_2_ (lower row) correlations for quenches to the SF regime [$U/J=0.5$, Fig. 3(a)], and to the MI regime, both slightly beyond the transition [$U/J=8$, Fig. 3(b)], and deep in the MI regime [$U/J=24$, Fig. 3(c)]. In all cases, at the notable exception of *G*_2_ deep in the MI phase [Fig. 3(c2), see discussion below], we find a twofold spike-like structure. The velocities *V*_m_ and *V*_CE_, extracted as before, are plotted on Fig. 3(d), showing similar results for *G*_1_ and *G*_2_. This is consistent with the prediction that these velocities are characterized by the spectrum, irrespective of the observable^27^.

In the SF regime, $U/J < {u}_{{\rm{c}}}$, the results compare very well with the predictions $2{V}_{\phi }^{\ast }$ and $2{V}_{{\rm{g}}}^{\ast }$ as found from the Bogoliubov dispersion relation (2) [see, respectively, the dashed blue and solid green lines on Fig. 3(d1 and d2)]. Quite surprizing, the agreement is fair up to the critical point where ${\gamma }_{{\rm{c}}}\simeq 1.6$, far beyond the validity condition of the Bogoliubov theory ($\gamma \ll 1$). In fact, when *U*/*J* increases from the mean-field regime, the momentum ${k}^{\ast }$ decreases down to the phonon regime, $k\ll \pi$, and the precise *k*-dependence of the dispersion relation beyond this regime becomes irrelevant. Moreover, the physics being dominated by long wavelength excitations, the lattice discretization in Eq. (1) may be disregarded and the BH model maps onto the continuous Lieb-Liniger model (For further details, see Supplemental Material. It contains information about the *t*-MPS calculations, the spreading of the one-body correlations (*G*_1_) in the meanfield regime and two-body correlations (*G*_2_) in the deep MI phase, as well as the mapping onto the Lieb-Liniger model). The latter is integrable by Bethe ansatz (BA)^51,52^. It yields the sound velocity ${V}_{{\rm{s}}}\simeq 2\overline{n}\sqrt{\gamma }(1-\sqrt{\gamma }/4\pi )$, to lowest order in the weak-*γ* expansion. Up to the critical point, the beyond-mean-field correction, $\sqrt{\gamma }/4\pi$, is less than 10%, which explains the good agreement between the numerics and the analytic formula. At the critical point, the numerical results for *V*_m_ and *V*_CE_ are consistent with the exact BA value $2{V}_{{\rm{s}}}\simeq 4.6$ (Close to the Mott-*U* critical point at $U/J=3.5$, we find ${V}_{{\rm{m}}}\simeq {V}_{{\rm{CE}}}\simeq 4.7$ (5.3) for the *G*_2_ (*G*_1_) correlation function, which agrees with the value of 2*V*_s_ within 2% (13%)).

The spreading velocities *V*_m_ and *V*_CE_ are continuous at the Mott-*U* transition, and do not show any critical behavior. Right beyond the critical point, they are still nearly equal and we can hardly distinguish two features from the numerics up to $U/J\simeq 6$. Deeper in the MI phase, however, we recover two distinct features and two different velocities. Contrary to the SF regime, here we find ${V}_{{\rm{m}}} > {V}_{{\rm{CE}}}$. These results are readily interpreted from the quasiparticle picture. Deep enough in the MI phase, $U/J\gtrsim 6$, the low-energy excitations are doublon-holon pairs, characterized by the dispersion relation^14,53^

3 $$2{E}_{k}\simeq \sqrt{{[U-2J(2\bar{n}+1)\cos (k)]}^{2}+16{J}^{2}\bar{n}(\bar{n}+1)\,{\sin }^{2}(k)}.$$

The comparison between the spreading velocities *V*_m_ and *V*_CE_ fitted from the *t*-MPS results and the characteristic values $2{V}_{\phi }^{\ast }$ and $2{V}_{{\rm{g}}}^{\ast }$, found from Eq. (3), yields a very good agreement within less than 5% for *G*_1_ and 9% for *G*_2_ [see Fig. 3(d1 and d2) respectively]. The quantitative agreement between the *t*-MPS results and the theoretical predictions for the *G*_1_ correlations persists up to arbitrary values of *U*/*J*. This validates the quasiparticle analysis also in the strong-coupling regime.

Yet, the *G*_2_ correlations behave differently. For intermediate interactions, $6\lesssim U/J\lesssim 9$, we find a twofold structure consistent with that found for *G*_1_. The signal for *G*_2_ blurs when entering deeper in the MI regime, and we are not able to identify two distinct features for $U/J\gtrsim 9$. To understand this behavior, one may resort on a strong-coupling ($U\gg J$) expansion of the correlation functions. In contrast to *G*_1_, the *G*_2_ function cannot be cast into the generic form analyzed in ref.^27^. Instead, combining Jordan-Wigner fermionization and Fermi-Bogoliubov theory^14^ (For further details, see Supplemental Material. It contains information about the *t*-MPS calculations, the spreading of the one-body correlations (*G*_1_) in the meanfield regime and two-body correlations (*G*_2_) in the deep MI phase, as well as the mapping onto the Lieb-Liniger model), one finds ${G}_{2}(R,t)\simeq -\,2|{g}_{2}(R,t){|}^{2}$ with

4 $${g}_{2}(R,t)\propto \frac{J}{U}\frac{R}{t}\,{\int }_{-\pi }^{+\pi }\,\frac{{\rm{d}}k}{2\pi }\{{e}^{i(2{E}_{k}t+kR)}+{e}^{i(2{E}_{k}t-kR)}\}.$$

For $U\gg 2(2\bar{n}+1)J$, the doublon-holon pair dispersion relation (3) reduces to $2{E}_{k}\simeq U-2(2\bar{n}+1)J\,\cos (k)$. Owing to the square modulus in the formula ${G}_{2}(R,t)\simeq -\,|{g}_{2}(R,t){|}^{2}$, we immediately find that the Mott gap *U* becomes irrelevant and we are left with the effective dispersion relation $2{\tilde{E}}_{k}\simeq -\,2(2\bar{n}+1)J\,\cos (k)$. On the one hand, the group velocity is not affected and we find the maximum value $2{V}_{{\rm{g}}}^{\ast }\simeq 2(2\bar{n}+1)J/\hslash$ at ${k}^{\ast }\simeq \pi /2$. The value $2{V}_{{\rm{g}}}^{\ast }=6J/\hslash$ found for $\bar{n}=1$ is in excellent agreement with the value of *V*_CE_ fitted from the *G*_2_ function deep in the MI phase, see Fig. 3(d2). On the other hand, the corresponding effective phase velocity vanishes, $2{\tilde{V}}_{\phi }^{\ast }\simeq 0$. This is consistent with the disappearance of the spike-like structure observed in the *t*-MPS calculations for *G*_2_ deep in the MI phase (More precisely, we find that in the vicinity of the CE both the real and imaginary parts of *g*_2_ display a series of static local maxima, consistently with $2{\tilde{V}}_{\phi }^{\ast }\simeq 0$. These local maxima are shifted by half a period and cancel each other when combined for constructing *G*_2_ (For further details, see Supplemental Material. It contains information about the *t*-MPS calculations, the spreading of the one-body correlations (*G*_1_) in the meanfield regime and two-body correlations (*G*_2_) in the deep MI phase, as well as the mapping onto the Lieb-Liniger model)). In addition, the first-order correction to the leading strong-coupling term, relevant for moderate values of *U*/*J*, sustains a double structure with ${V}_{{\rm{g}}}^{\ast }\ne {V}_{\phi }^{\ast }$. The latter is consistent with the observation of two distinct spreading velocities, ${V}_{{\rm{CE}}}\ne {V}_{{\rm{m}}}$, closer to the Mott-*U* transition (For further details, see Supplemental Material. It contains information about the *t*-MPS calculations, the spreading of the one-body correlations (*G*_1_) in the meanfield regime and two-body correlations (*G*_2_) in the deep MI phase, as well as the mapping onto the Lieb-Liniger model).

## Strongly Interacting Superfluid Regime

We finally consider the strongly interacting regime of the SF phase, corresponding to $\gamma \gg 1$ and $\bar{n}\notin {\mathbb{N}}$. In this regime, the Tomonaga-Luttinger liquid (TLL) theory accurately describes the low-energy physics of the BH model at equilibrium, including the Mott-*δ* transition, see for instance refs^30,54,55^. The TLL theory considers an effective harmonic fluid, characterized by a single characteristic velocity, namely the sound velocity *V*_s_.

**Figure 4:**
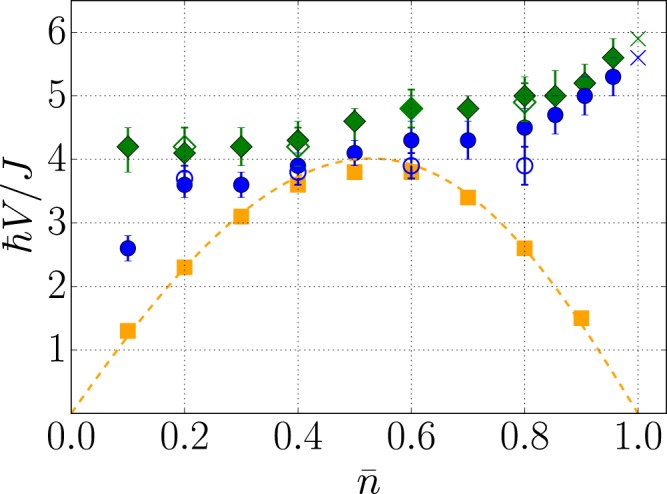
Twofold spreading of the *G*_2_ correlations in the strongly interacting SF regime for *U*/*J* = 50 and $0 < \bar{n} < 1$. Shown are the spreading velocities *V*_CE_ (green diamonds) and *V*_m_ (blue disks) fitted from the *t*-MPS simulations, together with twice the sound velocity 2*V*_s_ of the BH model as found from Bose-Fermi mapping (dashed orange line) and from MPS calculations (orange squares) (The sound velocity *V*_s_ has been computed by mapping the BH model to an equivalent spinless Fermi model^56,57^ (dotted orange line) and, independently, from the energy of the first excited state in exact MPS calculations (see orange squares), showing excellent agreement). Filled symbols correspond to the initial interaction parameter (*U*/*J*)_0_ = 1 and open symbols to (*U*/*J*)_0_ = 40. The crosses are linear extrapolations of *V*_CE_ and *V*_m_ to the Mott-*δ* transition at $\bar{n}=1$.

In contrast, our *t*-MPS simulations in the strongly interacting SF regime clearly show beyond TLL physics. We have computed the spreading of correlations for a large value of the after-quench interaction parameter, *U*/*J* = 50, and varying the filling $\bar{n}$ up to the Mott-*δ* transition at $\bar{n}=1$ [see pink arrow on Fig. 1(a)]. The spreading velocities *V*_CE_ (green diamonds) and *V*_m_ (blue disks), found from fits to the two-body correlation function *G*_2_(*R*, *t*), are shown on Fig. 4. They show clear deviations from twice the sound velocity of the BH model in the strongly interacting limit, $2{V}_{{\rm{s}}}\simeq (4J/\hslash )\,\sin (\pi \bar{n})\,[1-(8J/U)\,\cos (\pi \bar{n})]$ (orange dotted line and squares) (The sound velocity *V*_s_ has been computed by mapping the BH model to an equivalent spinless Fermi model^56,57^ (dotted orange line) and, independently, from the energy of the first excited state in exact MPS calculations (see orange squares), showing excellent agreement). Moreover, the emergence of two different characteristic velocities, ${V}_{{\rm{CE}}}\ne {V}_{{\rm{m}}}$, indicates that the TLL approach is insufficient to describe the spreading of correlations, even upon renormalization of the effective TLL parameters. Note that the two velocities become nearly equal in the vicinity of the Mott-*δ* transition and reach the value ${V}_{{\rm{CE}}}\simeq {V}_{{\rm{m}}}\simeq 6J/\hslash$. This is consistent with the disappearance of the twofold structure and the value found for *V*_CE_ deep in the MI phase at $\bar{n}=1$, see Fig. 3(d).

## Conclusions

In summary, working within the case study of the Bose-Hubbard chain and using a numerically-exact many-body approach, we have presented evidence of a universal twofold dynamics for the spreading of correlations. The latter is characterized by two distinct velocities, corresponding to the spreading of local maxima on the one hand and to the CE on the other hand. This has been found in all the phases of the model. Exceptions appear only in a few cases, for instance (i) for specific observables in specific regimes, or (ii) when the two velocities happen to be equal, as found at the Mott critical points for instance.

Our predictions are directly relevant to quench experiments on ultracold Bose gases in optical lattices, where the dynamics of one-body and two-body correlation functions can be observed on space and time scales comparable to our simulations^5–10,15,44^. Importantly, while in most experiments and numerics the CE is infered from the behavior of the correlation maxima, our results show that the two must be distinguished. This is expected to be a general feature of short-range systems and should be relevant to models other than the sole BH model.

Moreover, our study may be extended to long-range systems, such as spin models as realized in trapped-ion experiments^18,19^. While the notions of a maximum group velocity and phase velocity may break down in such systems, the mean-field theory also predicts a twofold dynamics^27^. In this case, it is characterized by the coexistence of super-ballistic and sub-ballistic signals. The results of the present paper suggest that the twofold structure of the correlation function may also survive in strongly correlated regimes for long-range systems. The demonstration of this effect would shed light on the still debated scaling of the light cone in long-range lattice models.

## Supplementary information

Supplemental material
