# Supplementary material for: Twofold correlation spreading in a strongly correlated lattice Bose gas
Source: Sci Rep. 2019 Mar 11;9:4135. doi: 10.1038/s41598-019-40679-3 (PMC6411744; doi:10.1038/s41598-019-40679-3)
Supplement: Supplementary file 1 — Supplemental material [file 41598_2019_40679_MOESM1_ESM.pdf]

Supplementary material for

Twofold correlation spreading in a strongly correlated lattice Bose gas

Julien Despres, Louis Villa, and Laurent Sanchez-Palencia
CPHT, Ecole Polytechnique, CNRS, Université Paris-Saclay, F-91128 Palaiseau, France
(Dated: February 19, 2019)

In this supplemental material, we give more details about several points discussed in the main paper. In Sec. S1, we discuss the time-dependent matrix-product state (t-MPS) simulations and the choice of the numerical parameters to ensure the convergence of the results in all the regimes of the Bose-Hubbard model considered in the main paper. In Sec. S2, we present t-MPS results for the spreading of the one-body correlation function G_1 in the mean-field superfluid (SF) regime. Section S3 briefly outlines the mapping from the 1D Bose-Hubbard model to the Lieb-Liniger model and gives the correspondance of the parameters. Finally, in Sec. S4 we discuss the strong-coupling expansion of the correlation function G_2 for unit filling, $\overline{n} = 1$, and discuss the suppression of its twofold structure deep in the Mott insulator (MI) phase.

S1. TIME-DEPENDENT MATRIX-PRODUCT STATE SIMULATIONS

The numerical results reported in the main paper are all obtained using the time-dependent density-matrix renormalization group approach (DMRG) with the matrix-product state representation (t-MPS approach) [1–3]. It yields numerically-exact results on both equilibrium and out-of-equilibrium properties of low dimensional lattice models. The approach resorts on the Schmidt expansion of the many-body wave function and permits to reduce the Hilbert space to a finite, relevant subset, provided the entanglement entropy remains sufficiently small. Owing to the area law [4, 5], it is optimal for 1D lattice models with a finite local Hilbert space in gapped phases, the entanglement of which remains finite in the thermodynamic limit. It also applies to gapless phases, although with more stringent numerical parameters (high-filling cut-off and the bond dimension). To validate the accuracy of our results in all phases of the BH model, a systematic study of the effect of these parameters has been performed.

Truncation of the local Hilbert space.— For the BH model considered in this work, the local Hilbert space is spanned by the Fock basis of number states, $|n_R\rangle$, where $n_R \in \mathbb{N}$, which is infinite. However, the probability distribution of the lattice-site occupation n_R decays faster than exponentially in both the SF and MI phases. Accurate results can thus be obtained by cutting off the local Hilbert space to some value n_{max}. It is important to note that, in some cases, the value of n_{max} needs to be significantly much larger than the average filling $\overline{n}$ and its fluctuations. This observation is consistent with analyses of truncated Bose-Hubbard models using quantum Monte Carlo simulations [6].

The SF mean-field regime, which corresponds to a high filling factor $\bar{n}$ and the gapless dispersion relation, has the most binding criteria. We found that a good estimator for n_{max} is given by the condition $1 - \sum_{n=0}^{n_{\text{max}}} P(n) \lesssim 10^{-2}$, where $P(n)$ is the probability that n bosons occupy a given lattice site. In the SF mean-field regime, the probability distribution is nearly Poissonian, $P(n) \simeq \bar{n}^n e^{-\bar{n}}/n!$. For instance, for the filling factor $\overline{n} = 5$ used for the data of Fig. 2, it yields $n_{\text{max}} \gtrsim 12$. For the strongly correlated SF regime at $\overline{n} = 1$ considered for Fig. 3(a), the density fluctuations are significantly suppressed and using the same condition as previously leads to $n_{\text{max}} = 5$. For the MI phase at $\bar{n} = 1$ and moderate values of U/J ($15 \geq U/J \geq u_{\text{c}}$) considered for Fig. 3(b), we kept $n_{\text{max}} = 5$. Deep in the MI phase ($U/J \geq 15$), truncating the local Hilbert space to $n_{\text{max}} = 2$, as used for Fig. 3(c) turns out to be sufficient. Finally, the strongly interacting SF regime is the easiest case from a numerical point of view. Owing to the low filling factor $\bar{n} < 1$ and the large value of the interaction parameter U/J, the above condition also yields $n_{\text{max}} = 2$, as used for Fig. 4. In all cases, we have checked that the numerics are converged for these values of n_{max}.

Bond dimension.— Within the MPS approach, the many-body state for a M-site lattice is represented in the tensor network form

$$|\Psi\rangle = \sum_{n_1, n_2, \ldots n_M} A^{n_1}[1]\, A^{n_2}[2] \ldots A^{n_M}[M]\, |n_1, n_2, \ldots, n_M\rangle\,, \tag{S1}$$

where n_j spans a local Hilbert space basis. For the BH model, it corresponds to a Fock basis truncated at n_{max}. For each value of n_j, the quantity $A^{n_j}[j]$ is a $\chi_{j-1} \times \chi_j$ matrix, where χ_j is the rank associated to the Schmidt matrix when applying the j-th singular value decomposition [2]. The bond dimension χ is defined as the maximum rank, $\chi = \max_j(\chi_j)$, $j \in [0 \ldots M]$. Note that for open-boundary conditions, the quantities $A^{n_1}[1]$ and $A^{n_M}[M]$ are actually a row vector and a column vector, respectively, *i.e.* $\chi_0 = \chi_M = 1$.

In the numerics, the maximum value of χ is chosen sufficiently large so that the truncation does not affect the results. In practice, the calculations are run for several values of χ up to convergence of the correlation function $G_1(R,t)$ or $G_2(R,t)$. The

required value of χ significantly depends on the regime and on the observable. In the following, we give the values used for the final results presented in the paper.

For the SF mean-field regime [Figs. 2(a) and S1], we used the values $\chi = 300$ and $\chi = 450$ for the G_2 and G_1 functions, respectively. The bond dimension used for G_1 is higher than the one for G_2 due to the long-range phase correlations already present at equilibrium. For the SF strongly correlated regime at $\overline{n} = 1$ [Fig. 3(a)], we used $\chi = 300$ for both correlation functions. A similar value of χ was considered for moderate values of U/J in the MI phase at $\bar{n} = 1$ [Fig. 3(b)]. Deep in the MI phase [Fig. 3(b)], the bond dimension can be significantly decreased and we consider $\chi = 100$. Finally, in the SF strongly interacting regime at $U/J = 50$, we found that the value $\chi = 100$ is enough.

S2. ONE-BODY CORRELATION FUNCTION $\mathbf{G_1(R, t)}$ IN THE MEAN-FIELD REGIME

In the analysis of the SF mean-field regime reported in the main paper, we focused on the two-body correlation function $G_2(R, t)$. We have also studied the one-body correlation $G_1(R, t)$ using the same t-MPS simulations. We found that the dynamics of the G_1 function shows a spike-like structure, similar to that found for the G_2 function. The values of the correlation edge (V_{CE}) and maxima (V_{m}) velocities agree with those found for the G_2 function within less than 10%. Figure S1 shows an example, for the quench from $(U\overline{n}/J)_0 = 1$ to $U\overline{n}/J = 0.5$, and $\bar{n} = 5$. The fits to the correlation edge and to the maxima yield the velocities $V_{\text{CE}} = (4.4 \pm 0.3)\, J/\hbar$ and $V_{\text{m}} = (3.3 \pm 0.2)\, J/\hbar$, in excellent agreement with the corresponding values found from the dynamics of the G_2 function, see Fig. 2(b).

The agreement between the spreading velocities for different correlation functions was found in all regimes, see for instance Figs. 3(d1) and (d2). It is consistent with the prediction that these velocities are characteristic of the excitation spectrum and not on the details of the correlation function [7]. Note, however, that the full space-time dependence of the signal depends on the correlation function. In general, we found that the signal for G_1 is less sharp than for G_2. This may be attributed to the long-range phase correlations present in the initial state, which blur the correlation function [8].

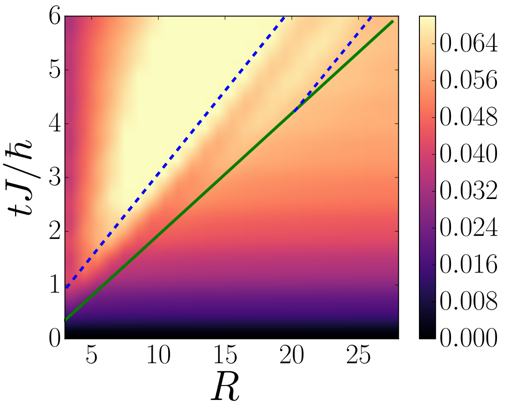

Figure S1: Spreading of the one-body correlation function $G_1(R, t)$ for a global quench in the SF mean-field regime from $(U/J)_0 = 0.2$ to $U/J = 0.1$ and $\bar{n} = 5$. The solid-green and dashed-blue lines are fits to the CE and maxima, respectively.

S3. MAPPING ON THE 1D LIEB-LINIGER MODEL

In the long-wave length regime, the lattice discretization of the Bose-Hubbard (BH) may be disregarded. The BH model then maps onto the continuous-space Lieb-Liniger (LL) model,

$$\hat{H} = \frac{\hbar^2}{2m}\left[-\sum_{i=1}^{N}\frac{\partial^2}{\partial x_i^2} + c\sum_{i\neq j}\delta(x_i - x_j)\right]. \tag{S2}$$

It describes a one-dimensional gas of N bosons of mass m with contact interactions, characterized by the interaction strength $c > 0$. The correspondance between the parameters of the BH and LL models is found by discretizing the LL model, Eq. (S2), on the length scale defined by the lattice spacing a. It yields $J = \hbar^2/2ma^2$ and $U = \hbar^2 c/ma$. The density of the LL model is $\rho \equiv N/L = \overline{n}/a$, where $\overline{n}$ is the number of bosons per lattice site (filling) and L is the system size.

The LL Hamiltonian is exactly solvable by Bethe ansatz [9, 10]. All the thermodynamic quantities at zero temperature can be written as universal functions of the Lieb-Liniger parameter $\gamma = c/\rho$ and the dimensionless quantity $e(\gamma) = E_0/Nn^2$, where E_0 is the ground state energy. For instance, the macroscopic sound velocity [10] reads as

$$v_{\mathrm{s}} \equiv \sqrt{\frac{L}{m\rho}\left.\frac{\partial^2 E_0}{\partial L^2}\right|_{N,S}} = \frac{\hbar\rho}{m}\sqrt{3e(\gamma) - 2\gamma e'(\gamma) + \frac{1}{2}\gamma^2 e''(\gamma)}. \tag{S3}$$

Using the small γ expansion, $e(\gamma) = \gamma\left[1 - (4/3\pi)\sqrt{\gamma}\right]$, one then finds

$$v_{\mathrm{s}} = \frac{\hbar\rho}{m}\sqrt{\gamma}\left(1 - \sqrt{\gamma}/4\pi\right), \tag{S4}$$

valid in the weakly-interacting regime, $\gamma \ll 1$. Finally, using the correspondance between the parameters of the BH and LL models, one finds

$$V_{\mathrm{s}} \equiv v_{\mathrm{s}}/a = \frac{2J\overline{n}}{\hbar}\sqrt{\gamma}\left(1 - \sqrt{\gamma}/4\pi\right) \tag{S5}$$

and $\gamma = U/2J\overline{n}$.

S4. TWO-BODY CORRELATION FUNCTION $\mathbf{G_2(R,t)}$ IN THE MOTT-INSULATING PHASE

In order to explain the suppression of the twofold structure for the two-body correlations deep in the Mott insulator phase (MI; $U \gg J$ and $\overline{n} = 1$), we compute the function $G_2(R,t)$, working along the lines of Ref. [11]. Considering the manifold of doublon-holon pairs and mapping the resulting Hamiltonian into a fermionic one, the two-body correlation function may be written as

$$G_2(R,t) \simeq -2\left(|g_2(R,t)|^2 + |\bar{g}_2(R,t)|^2\right), \tag{S6}$$

with

$$g_2(R,t) \sim \frac{J}{U}\frac{R}{t}\int_{-\pi}^{+\pi}\frac{\mathrm{d}k}{2\pi}\left\{\mathrm{e}^{i(2E_k t + kR)} + \mathrm{e}^{i(2E_k t - kR)}\right\}, \tag{S7}$$

$$\bar{g}_2(R,t) \sim \left(\frac{J}{U}\right)^2\int_{-\pi}^{+\pi}\frac{\mathrm{d}k}{2\pi}\sin^2(k)\left\{\mathrm{e}^{i(2E_k t - kR)} + \mathrm{e}^{-i(2E_k t + kR)}\right\} \tag{S8}$$

and the excitation spectrum is $2E_k \simeq \sqrt{\left[U - 2J(2\overline{n}{+}1)\cos(k)\right]^2 + 16J^2\overline{n}(\overline{n}{+}1)\sin^2(k)}$, see Eq. (3).

Quench deep into the Mott insulator phase.— For a quench, very deep in the MI phase, $U \gg J$, the second right-hand-side term in Eq. (S6) is much smaller than the first one and the former can be neglected. Using Eq. (S7), it yields explicitly for $G_2(R,t) \simeq -2|g_2(R,t)|^2$,

$$G_2(R,t) \sim -2\left(\frac{J}{U}\right)^2\left(\frac{R}{t}\right)^2\left|\int_{-\pi}^{\pi}\frac{\mathrm{d}k}{2\pi}\left\{\mathrm{e}^{i(2E_k t + kR)} + \mathrm{e}^{i(2E_k t - kR)}\right\}\right|^2 \tag{S9}$$

Moreover, the excitation spectrum may be expanded in powers of J/U. Up to first-order, it yields $2E_k \simeq U - 2J(2\overline{n}{+}1)\cos(k)$. The gap term e^{iUt} can then be factorized in the two terms under the integral in Eq. (S9) and disappears due to the square modulus. Introducing the effective excitation spectrum $2\tilde{E}_k = -2J(2\overline{n}+1)\cos(k)$, we then find $G_2 \simeq -2|g_2(R,t)|^2$ with

$$g_2(R,t) \sim \frac{J}{U}\frac{R}{t}\int_{-\pi}^{\pi}\frac{\mathrm{d}k}{2\pi}\left\{\mathrm{e}^{i\left(2\tilde{E}_k t + kR\right)} + \mathrm{e}^{i\left(2\tilde{E}_k t - kR\right)}\right\}. \tag{S10}$$

The integral may be evaluated using the stationary phase approximation. In the infinite time and distance limit along the line $R/t = \mathrm{cst}$, the integral in Eq. (S10) is dominated by the momentum contributions with a stationary phase (sp), *i.e.* $\partial_k(2\tilde{E}_k t \pm kR) = 0$ or, equivalently, $2\tilde{V}_{\mathrm{g}}(k_{\mathrm{sp}}) = \pm R/t$ where $\tilde{V}_{\mathrm{g}} = \partial_k\tilde{E}_k$ is the group velocity of the effective excitation spectrum. Since the latter is upper bounded by the value $\tilde{V}_{\mathrm{g}}^* = \max(\tilde{V}_{\mathrm{g}}) = J(2\overline{n}+1)$, it has a solution only for $R/t < 2\tilde{V}_{\mathrm{g}}^*$. We then find

$$g_2(R,t) \sim \frac{J}{U}\frac{\tilde{V}_{\mathrm{g}}(k_{\mathrm{sp}})}{\left(|\partial_k^2\tilde{E}_{k_{\mathrm{sp}}}|t\right)^{1/2}}\left[\cos\left(2\tilde{E}_{k_{\mathrm{sp}}}t - k_{\mathrm{sp}}R + \sigma\frac{\pi}{4}\right) + i\sin\left(2\tilde{E}_{k_{\mathrm{sp}}}t - k_{\mathrm{sp}}R + \sigma\frac{\pi}{4}\right)\right]. \tag{S11}$$

with $\sigma = \mathrm{sgn}\left(\partial_k^2 \tilde{E}_{k_{\rm sp}}\right)$. For both the real and imaginary parts of $g_2(R,t)$, the correlations are activated ballistically at the time $t = R/2\tilde{V}_{\rm g}^*$. It defines a linear correlation edge (CE) with velocity $V_{\rm CE} = 2\tilde{V}_{\rm g}^*$. In addition, Eq. (S11) also yields a series of local maxima, defined by the equation $2\tilde{E}_{k_{\rm sp}}t - k_{\rm sp}R = \mathrm{cst}$. In the vicinity of the CE cone, these maxima (m) propagate at the velocity $V_{\rm m} = 2\tilde{V}_\varphi^* = 2\tilde{E}_{k^*}/k^*$, *i.e.* twice the phase velocity at the maximum of the group velocity, k^*.

Hence, the real and imaginary parts of $g_2(R,t)$ both display a twofold structure with a CE velocity $2\tilde{V}_{\rm g}^* = 2J(2\bar{n}+1)$ and a velocity of the maxima $2\tilde{V}_\varphi^* = 0$, as shown on Figs. S2(a) and (b). In contrast, $G_2(R,t)$, does not display the twofold structure. This is because it is the sum of the squares of the two latter contributions [see Eq. (S11)], which are shifted by half a period and cancel each other. It thus gives a single cone structure, characterized by the sole CE velocity $2\tilde{V}_{\rm g}^*$, as shown on Fig. S2(c).

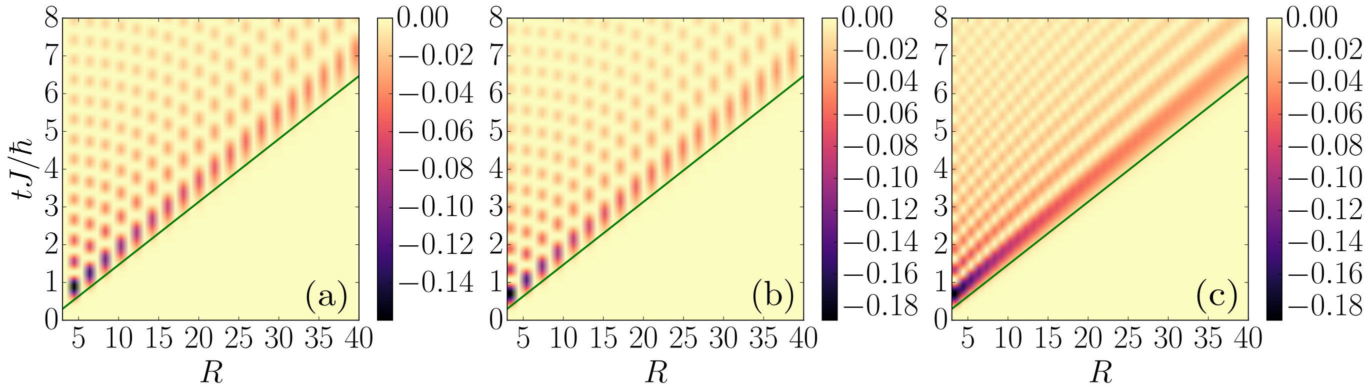

Figure S2: Analysis of the space-time correlation pattern of $G_2(R,t)$ via $g_2(R,t)$ [see Eq. (S10)] at $\bar{n} = 1$ for a global quench confined deep into the Mott-insulating phase starting from a pure Mott state $(U/J)_0 \to \infty$. Analytical expression, owing to prefactors, of (a) $-\Re^2\left[g_2(R,t)\right]$ (b) $-\Im^2\left[g_2(R,t)\right]$ (c) sum of the two contributions shown at Fig. (a) and (b). The solid green line corresponds to the theoretical CE velocity characterized by $2\tilde{V}_{\rm g}^* = 2J(2\bar{n}+1)$. On Fig. (c), the first extremum propagates with the same velocity as the one associated to the CE.

Quench into the Mott insulator phase for moderate U/J.— For moderate values of U/J, still in the MI phase, the second term in the right-hand-side of Eq. (S6), $|\bar{g}_2(R,t)|^2$, becomes relevant. Using again the stationary-phase approximation for $\bar{g}_2(R,t)$, we find

$$\bar{g}_2(R,t) \sim \left(\frac{J}{U}\right)^2 \frac{\sin^2(k_{\rm sp})}{\left(|\partial_k^2 E_{k_{\rm sp}}|t\right)^{1/2}} \cos\left(2E_{k_{\rm sp}}t - k_{\rm sp}R + \sigma'\frac{\pi}{4}\right) \tag{S12}$$

with $\sigma' = \mathrm{sgn}\left(\partial_k^2 E_{k_{\rm sp}}\right)$ and E_k the excitation spectrum given at Eq. (3). Using the same argument as above, we find that $\bar{g}_2(R,t)$ shows a twofold structure characterized by, now, the CE velocity $2V_{\rm g}^* = 2\max\left(\partial_k E_k\right)$ but the velocity of the maxima $2V_\varphi^* = 2E_{k^*}/k^* \neq 0$. Since there is a single contribution here, the quantity $|\bar{g}_2(R,t)|^2$ displays a twofold structure with the same characteristic velocities. More precisely, both the length and time scales of the oscillations are divided by two but the velocities are not affected.

For a quench into the MI phase at a moderate value of U/J, both $|g_2(R,t)|^2$ and $|\bar{g}_2(R,t)|^2$ contribute to the two-body correlation function $G_2(R,t)$. While the $|g_2(R,t)|^2$ contribution is characterized by the sole CE velocity $2V_{\rm g}^*$, the $|\bar{g}_2(R,t)|^2$ contribution provides the double structure observed on G_2 for $6 < U/J < 10$ in the t-MPS calculations.

---

[1] U. Schollwöck, *The density-matrix renormalization group*, Rev. Mod. Phys. **77**, 259 (2005).
[2] U. Schollwöck, *The density-matrix renormalization group in the age of matrix product states*, Ann. Phys. (NY) **326**, 96 (2011).
[3] M. Dolfi, B. Bauer, S. Keller, A. Kosenkov, T. Ewart, A. Kantian, T. Giamarchi, and M. Troyer, *Matrix product state applications for the ALPS project*, Comput. Phys. Commun. **185**, 3430 (2014).
[4] M. M. Wolf, F. Verstraete, M. B. Hastings, and J. I. Cirac, *Area laws in quantum systems: Mutual information and correlations*, Phys. Rev. Lett. **100**, 070502 (2008).
[5] J. Eisert, M. Cramer, and M. B. Plenio, *Colloquium: Area laws for the entanglement entropy*, Rev. Mod. Phys. **82**, 277 (2010).
[6] V. A. Kashurnikov, A. V. Krasavin, and B. V. Svistunov, *Zero-point phase transitions in the one-dimensional truncated bosonic Hubbard model and its spin-1 analog*, Phys. Rev. B **58**, 1826 (1998).
[7] L. Cevolani, J. Despres, G. Carleo, L. Tagliacozzo, and L. Sanchez-Palencia, *Universal scaling laws for correlation spreading in quantum systems with short- and long-range interactions*, Phys. Rev. B **98**, 024302 (2018).

[8] S. Bravyi, M. B. Hastings, and F. Verstraete, *Lieb-Robinson bounds and the generation of correlations and topological quantum order*, Phys. Rev. Lett. **97**, 050401 (2006).
[9] E. H. Lieb and W. Liniger, *Exact analysis of an interacting Bose gas. I. The general solution and the ground state*, Phys. Rev. **130**, 1605 (1963).
[10] E. H. Lieb, *Exact analysis of an interacting Bose gas. II. The excitation spectrum*, Phys. Rev. **130**, 1616 (1963).
[11] P. Barmettler, D. Poletti, M. Cheneau, and C. Kollath, *Propagation front of correlations in an interacting Bose gas*, Phys. Rev. A **85**, 053625 (2012).
